# Implementation of health literacy training for clinicians in a federally qualified health center

**DOI:** 10.1016/j.pecinn.2022.100083

**Published:** 2022-09-14

**Authors:** Jacqueline Naperola-Johnson, Jose Gutierrez, Kathryn Doyle, Julie Thompson, Cristina Hendrix

**Affiliations:** a School of Nursing, Duke University school of nursing, Durham, United States; b Philip R. Lee Institute for Health Policy Studies, University of California San Francisco, San Francisco, United States

**Keywords:** Health literacy, Clear communication, Clinicians' training

## Abstract

**Objective:**

The objective of this quality improvement project was to evaluate the effectiveness of a succinct health literacy training for providers at a demanding federally qualified health center.

**Methods:**

One group, pretest-posttest design was used to measure for a change in knowledge regarding the effects of limited health literacy, a change in self-reported measure of routine screening for limited health literacy and a change in self-reported utilization of patient-centered communication techniques.

**Results:**

The average percentage of correct responses on the Health Literacy Knowledge Check showed significant improvement from 23.6% (SD = 18.1%) to 63.9% (SD = 25.3%), *p* < .001. There were no significant changes in median responses at pre- and post-intervention for self-reported use of screening and communication techniques (all *p* > .05).

**Conclusion:**

This brief training was effective at improving participants' knowledge of health literacy but did not improve use of recommended communication techniques or screening for health literacy. The results suggest that emphasizing a universal precautions approach to health literacy may be more effective with participants who work in high-volume clinics.

**Practice implications:**

For high-volume clinics, a brief training may improve participants' knowledge but does not increase use of actual communication techniques based on self-report.

## Highlights

• A brief training on health literacy is often necessary in high-volume clinics
• Brief training may improve providers' knowledge of health literacy but role play is recommended to help improve behavior
• Recommend universal precautions method to simplify patient approach and create intervention persistence

## 1 Introduction

The Center for Disease Control [1] defines health literacy (HL) as “the degree to which individuals have the ability to find, understand, and use information and services to inform health-related decisions and actions for themselves and others.” It is estimated, however, that 90 million people, or nearly half of American adults, do not possess the level of HL that will allow them to understand how to navigate the health care system [2]. On an individual level, when comparing expenditure for patients with limited health literacy (LHL) to those with satisfactory HL, the additional burden ranges from $143 to $7798 [3]. On a system level, this equates to 3–5% of total health care costs [3]. One contributing factor to these costs is a system that requires patients to read at almost every point along the healthcare spectrum. This non-user-friendly structure may produce fear and embarrassment which inhibit patients from seeking clarification on treatment instructions or medical advice, thus resulting in further costly effects [4]. Beyond the financial burden, LHL is associated with poorer health outcomes and a decrease in overall health-related quality of life (HRQL) [[5], [6], [7], [8], [9]].

With these negative ramifications, HL is often still an overlooked component of routine medical care or overestimated by healthcare providers and clinicians upon intuitive assessment [[10], [11], [12]]. Often assessment of HL is not conducted as part of clinic visits or before disease management is discussed yet it is a fundamental component of patient-centered communication (PCC), or the “oral exchange” between providers and patients that depends on mutual oral and aural literacy [6]. Executed successfully, PCC increases patient satisfaction, engagement, adherence to medical advice and subsequently improves overall health outcomes [13].

A patient's health literacy is not information that is readily available to providers as they conduct visits. There are, however, risk factors that may indicate a patient will struggle with health literacy. These risk factors include poverty, race and ethnicity, being an immigrant, English being a second language, level of education (high school or lower), and being older than 65 [[14], [15], [16]]. Garcia and colleagues found a prevalence of 64.9% limited health literate patients in their sample of older Hispanics patients and a prevalence of 31% LHL in their caregivers [17]. Immigrants, particularly noncitizens and undocumented, are less likely to have health insurance and have a lower use of health services [18]. Providers who work in under-resourced clinics are often uniquely situated to treat patients with multiple risk factors and through this have greater opportunity to build healthy and dependable patient-provider relationships.

Healthcare providers routinely assume that the information provided to patients and their families is well-understood [19]. This is not always the case, however, and may lead to apparent non-adherence or other unintended consequences. Accurate assessment of HL enables healthcare providers to align medical information with a patient's HL skills. There are a variety of tools to assess health literacy. While some are labor intensive and not feasible for regular use in a demanding clinic setting, there are also a number of short, validated tools that are convenient for rapid assessment. Some examples of these are the Brief Health Literacy Screen (BRIEF), the Short Test of Functional Health Literacy in Adults (S-TOFHLA), and the Rapid Estimate of Adult Literacy in Medicine (REALM) [[20], [21], [22], [23]].

The objective of this quality improvement project was to study the effectiveness of an abbreviated training for providers at a high-volume multi-ethnic, multi-lingual federally qualified health center (FQHC) in Los Angeles County. Many of the patients at the FQHC have one or more risk factors for LHL further increase the importance of studies related to this subgroup. The training included evidence-based information concerning the topic of health literacy (HL), its impact on disease management and health-related outcomes, the benefit of employing HL assessments to guide patient-provider communication and communication techniques that may be utilized to engage patients with LHL.

## 2 Methods

### 2.1 Study design

One group, pretest-posttest design was used to measure for a change in knowledge regarding the effects of LHL, a change in self-reported measure of routine HL screening and a change in self-reported utilization of patient-centered communication techniques.

### 2.2 Setting and sample

The setting for this quality improvement project was a FQHC comprised of six locations in Los Angeles County. The FQHC is a non-profit clinic that provides services for those who qualify for the My Health LA (MHLA) program, a no-cost health care program provided by the Los Angeles County Department of Health Services for those who reside in the county but do not have access to health insurance [24]. Due to the nature of the MHLA program, the FQHC services a diverse patient population, many who identify as undocumented immigrants. Consequently, ensuring effective communication with patients navigating a complex healthcare system is critical to quality patient care.

Participants of this quality improvement project were 23 clinicians who attended the center's monthly provider meeting. The health center utilizes monthly provider meetings to provide general clinic updates and deliver various trainings to their clinicians. Meeting attendance and the included HL training were compulsory however participants were free to decline the anonymous pre- and post-training assessments. There was no demographic data nor data on number of years of practice for the participants to ensure a sense of anonymity with the surveys. The providers were a mix of medical doctors (MDs), doctors of osteopathy (DOs), nurse practitioners (NPs), and physician's assistants (PAs). They represented a range of specialties within the organization which include family medicine, pediatric medicine, and women's health. There was no information about the training provided prior to session. Informed consent for participants was not acquired from clinicians for participation in this training.

Due to SARS-CoV-2 restrictions the monthly meeting was moved to a virtual format. The training was then modified to only include the didactic teaching but eliminated the in-person role-play session that was initially planned. The project was not reviewed by an ethical committee.

### 2.3 Intervention

One training session was conducted with clinicians in October 2020. The training was completely developed and delivered by the authors of the study in a lecture-style presentation format allowing for questions or comments throughout and at the end of the presentation. The training was not pilot-tested and was delivered for the first time for this study. Following the lecture-style presentation the training was originally supposed to include a short session where providers paired off and engaged in patient-provider role scenarios utilizing different tools that had been discussed. Due to a wave of the SARS-CoV-2 pandemic, the entire provider meeting pivoted from an in-person to a video conferencing platform. As a result, providers attending the training needed time to access the pre-assessment online. As the training was scheduled for 30 min, allotting time for the pre-assessment, technical difficulties and the lecture left no time for the role-play scenario, and it was eliminated.

To garner baseline knowledge and habits, participants completed the Health Literacy Knowledge Check (HLKC) (Appendix A) and the Health Literacy Screening Frequency and Communication Techniques Survey (HLSFCTS) (Appendix B) prior to the training. The The thirty-minute virtual training session was then conducted. Participants subsequently completed a post-training HLKC. At one-month post-intervention participants also completed a follow-up HLSFCTS.

### 2.4 Health literacy training description

The training encompassed three key foci:

1. Background information on HL: population percentages, HL red flags, HL effects on disease management and health-related outcomes
2. Use of a short, validated tool to assess HL (BRIEF)
3. Patient-centered communication techniques that support patients with LHL

The training provided was created by the authors of this study and emphasized numerous evidence-based concepts found in current health literacy literature [4,6,11,19,23,[25], [26], [27]]. Some examples of these concepts include common risk factors for LHL such as race, poverty, and educational attainment; the overall percentage of the population with reading illiteracy; a description of subjective versus objective measures of literacy along with an example of a subjective screening tool (BRIEF); information regarding the routine overestimation of patient HL by providers and its negative consequences; and validated communication techniques [28]. The communication techniques that were discussed were:

• The use of plain language in exchange for medical jargon
• The interactive communication loop, or the “teach-back” method which encourages the patient to teach the information back using their own words
• Use of open-ended questions such as “What questions do you have?” as opposed to “Do you have any questions?”
• Placing the onus of clear communication on the health care provider and not the patient

### 2.5 Outcome measures

Provider knowledge regarding the topic of HL and its subsequent effects on health-related outcomes was measured comparing the scores on the HLKC pre-intervention and immediately post-intervention. The HLKC is a 7-item knowledge test composed of true-false and multiple-choice questions based on the concepts of HL covered during the training. This tool was created by the authors of the study and is not validated. This also served as the fidelity measure to determine whether the training operated as intended.

Provider utilization of communication techniques and screening tools was measured by comparing answers on the HLSFCTS immediately before the training and again one month after to provide long-term data on any changes in the use of a screening tool or communication techniques. This was a doctoral project and the one-month post-test allotted enough time to garner long-term data while allowing the lead author time to run statistical tests and create a doctoral defense. The HLSFCTS was created by the authors of this study based on similar Likert-style screenings that were utilized in other studies to assess pre-post self-reported behaviors by providers. [29,30]. The HLSFCTS is a self-report survey containing 5-point, Likert-style responses (1 - Always, 2 - Often, 3 - Sometimes, 4 - Rarely, 5 - Never) to the following statements: (1) I screen patients for limited health literacy, (2) I avoid using medical jargon when talking with patients and define unavoidable jargon in lay terms, (3) I use the interactive communication loop, or the teach-back method, when seeing patients (4) I elicit questions from patients using a patient-centered, open-ended approach (e.g. “what questions do you have?” rather than “do you have any questions?”), (5) I put the responsibility of clear communication on myself as the clinician (e.g. “I've just said a lot of things. To make sure I did a good job and explained things clearly, can you describe to me….?”). The one-month follow up survey also included the question “What difficulties, if any, do you encounter when trying to implement health literacy communication techniques with patients?” This tool is not validated.

The HLKC was only completed before and immediately after the training because the knowledge about LHL presented in the training was used as an emphasis on the importance of screening for HL and using patient-centered communication. There are also several studies on health literacy education that measure participants' intentions and planned behaviors related to HL communication techniques [22,[24], [25]]. This project did not measure immediate post-intervention intention to use communication techniques or screening tools.

### 2.6 Data analysis

Data analysis was performed with IBM SPSS version 27. For the knowledge quiz, independent samples *t*-test was used to examine the average percentage of correct responses. Individual items were examined using descriptive statistics. For Likert Items, since data were independent, Mann Whitney *U* tests were used. Response values ranged from 1 (Always) to 5 (Never).

## 3 Results

The average percentage of correct responses on the HLKC showed significant improvement from 23.6% (SD = 18.1%) to 63.9% (SD = 25.3%), *p* < .001. Individual items were examined using descriptive statistics and are displayed in Table 1. Improvement occurred for all seven items.

**Table 1:** Pre- and post-intervention knowledge items.

Item	Correct Responses			
	Pre (*n* = 23)		Post (*n* = 17)	
	*n*	*%*	*n*	*%*
The majority of people in the United States with limited health literacy are white, native-born Americans	8	34.8	11	64.7
Adults with a high school diploma typically read at what grade level?	4	17.4	11	64.7
The average reading level of US adults is	4	17.4	9	52.9
Reading literacy is a direct indicator of health literacy	4	17.4	9	52.9
Which of the following is not a red flag for limited health literacy?	5	21.7	10	58.8
Patients understand and retain how much of what a provider is telling them on average?	5	21.7	16	94.1
Being a female is a risk factor for limited health literacy	8	34.8	10	58.8

HLSFCTS Likert-scale self-assessment items response values ranged from 1 (Always) to 5 (Never) and the results are displayed in Table 2. Lower scores are indicative of improvement for self-assessment items. There were no significant changes in median responses at pre- and post-intervention (all *p*s > 0.05).

**Table 2:** Self-assessment comparison at Pre- and Post-Intervention.

Survey Item	Pre (*n* = 23)		Post (*n* = 6)		*P*
	*Mdn*	*IQR*	*Mdn*	*IQR*	
I screen patients for limited health literacy	3	3–4	3	2–3	0.056
I avoid using medical jargon when talking with patients and define unavoidable jargon in lay terms	2	1–2	2	1–2	0.948
I use the interactive communication loop, or the teach-back method, when seeing patients	2	2–3	2.5	1.75–3.25	0.954
I elicit questions from patients using a patient-centered, open-ended approach (e.g., “what questions do you have?” rather than “do you have any questions?”)	2	2–3	2	1–3	0.275
I put the responsibility of clear communication on myself as the clinician (e.g., “I've just said a lot of things. To make sure I did a good job and explained things clearly, can you describe to me….?”)	3	2–3	2	2–3	0.356

*Note*. IQR is presented as P _25_ – P _75_.

In response to the post-assessment question, “What difficulties, if any, do you encounter when trying to implement health literacy communication techniques with patients?” all participants (*n* = 6) reported lack of time as the biggest roadblock. One participant also put educational background and language barriers as a difficulty.

## 4 Discussion

### 4.1 Discussion

The purpose of this quality improvement project was to evaluate the effectiveness of a succinct training on health literacy in a demanding FQHC. There are several studies that evaluate the effects of health literacy education in various settings [[25], [26],[29], [30], [31], [32]] yet time-intensive trainings are often not feasible in the under-resourced, high-volume health centers that care for underserved populations. With the high number of FQHCs across the country that provide healthcare for patients with LHL, curating an effective training that educates healthcare providers on techniques to address health literacy disparity is crucial to improving PCC, HRQL and health-related outcomes.

Pretest-posttest outcome measures for this project showed significant improvement for all knowledge items immediately post-training. There was not, however, a significant change in any self-assessment measures of participant behaviors. The results of the self-assessment measures may be related to the limitations in the project's methodology. Due to a wave of the SARS-CoV-2 pandemic, the role-play portion of the training was eliminated. The authors feel that this was a key component of the training. It is suggested that future trainings on HL may benefit from the addition of role-play to increase comfort in using various communication techniques. Another limitation is sample size. The participants were only the pool of providers who work for the health center. The size is further reduced because completion of the assessment tools was not mandatory. As such, the number of participants (*n* = 23) who attended and completed the pre-training assessment was higher than those who completed the immediate post-assessment (*n* = 17). Even less (*n* *=* 6) completed the final one-month post-assessment. In part this may be because the assessment was transferred to an online survey when the training was moved from in-person to virtual. Filling out an assessment in person rather than virtually may have prompted more participation.

There is a wealth of information about health literacy screening tools. Some are better utilized in research while others more easily align with the flow of clinical practice. This training provided an example of a screening tool, the BRIEF, in measuring for LHL [21,23]. In the absence of the role-play in the educational training providers were not afforded the opportunity to practice screening a patient and then utilizing the recommended approaches to communicate. Providers who did not go on to use the screening tool may have subsequently omitted using the discussed communication strategies as there was no designated low screening score highlighting a patient's LHL. This likely lead to engaging patients in the same way providers always had and foregoing the use of both the screening and the communication techniques that were discussed.

One way to address this is to exchange the universal screening of health literacy, highlighted during this training, for the “universal precautions” approach [4,11,29,[33], [34]]. The universal precautions approach negates the need for screening by assuming that all patients, regardless of education, may have difficulty understanding health information. Emphasizing a universal precautions method removes the screening process and creates a more homogenous approach to patient communication. In a demanding clinic where time is scarce, stressing a streamlined approach might save providers the burden of categorizing patients while also providing an opportunity to practice and improve communication techniques with each patient encounter.

Finally, the HLKC was only administered immediately following the training. The information about HL provided in the presentation was intended to increase participants' awareness of LHL and augment intention to use patient-centered communication. Evaluating their knowledge levels over a longer period would provide more information on whether participants maintained this knowledge in future practice.

### 4.2 Innovation

The focus of this innovation was evaluating the effectiveness of a training delivered to healthcare providers on the successful exchange of information between clinicians and LHL patients that quality and cost-effective health care necessitates. Prior studies educating providers on measures to address health literacy that demonstrated intervention persistence, or effecting a lasting change, utilized lengthier trainings. To the authors' knowledge no interventions to date evaluate the effectiveness of a briefer instruction time for health care providers working in under-resourced settings with patients highly vulnerable to limited health literacy. It can be difficult to generate lasting transformation in these demanding, high-volume clinics that don't allow for lengthy training opportunities and yet providers in these clinics are particularly well-positioned to have greater impact in promoting health equity for the most vulnerable populations. Finding successful ways to deliver information in a succinct but transformative way is crucial to improving patient care in such clinics where it is needed most.

There are generally two methods to screen for limited health literacy: a validated screening tool or a universal precautions approach. The educational session with clinicians utilized a validated screening tool which added complexity to an already brief training period. This was evident when clinicians in the community health center who participated cited time as the leading factor for why they felt unable to both implement tools to assess and then address health literacy in their patients. This project attempted to create intervention persistence through a brief educational session for clinicians with limited time resource for clinical patient care and supplementary training. While the results did not demonstrate long-term behavior change it is the first of its kind and establishes the importance of creating simpler guidelines for brief instruction times which may result in long-term transformation.

### 4.3 Conclusion

This brief virtual training showed significant improvement in baseline knowledge of health literacy. There was no significant change in self-reported use of health literacy screening tools or self-assessment of implementation of communication techniques. This may be due to the limitations of the training as well as the methodology.

## Funding

The authors have no funding sources to disclose.

I confirm all patient/personal identifiers have been removed or disguised so the patient/person(s) described are not identifiable and cannot be identified through the details of the story.

## Declaration of Competing Interest

The authors report no conflicts of interest.

## Appendix A

Health Literacy Knowledge Check

1. The majority of people in the United States with limited health literacy are white, native-born Americans
  a. True
  b. False
2. Adults with a high school diploma typically read at what grade level?
  a. First-year college level
  b. 11th–12th grade level
  c. 9th–10th grade level
  d. 7th–8th grade level
  e. 5th–6th grade level
3. The average reading level of US adults is
  a. College level
  b. 11th–12th grade
  c. 9th–10th grade
  d. 7th–8th grade
  e. 5-6th grade
4. Reading literacy is a direct indicator of health literacy
  a. True
  b. False
5. Which of the following is not a red flag for limited health literacy?
  a. Patient over-engages to compensate for lack of knowledge
  b. Incomplete medical history or many items checked as “no”
  c. Takes instructions literally to avoid mistakes
  d. Patients make excuses when asked to read or fill out forms
  e. Signs of nervousness, confusion, frustration or indifference
6. Patients understand and retain how much of what a provider is telling them on average?
  a. 40%
  b. 50%
  c. 60%
  d. 70%
  e. 80%
7. Being a female is a risk factor for limited health literacy
  a. True
  b. False

## Appendix B

Health Literacy Screening Frequency and Communication Techniques Survey

1. I screen patients for limited health literacy
1. Always
2. Often
3. Sometimes
4. Rarely
5. Never
2. I avoid using medical jargon when talking with patients and define unavoidable jargon in lay terms
1. Always
2. Often
3. Sometimes
4. Rarely
5. Never
3. I use the interactive communication loop, or the teach-back method, when seeing patients
1. Always
2. Often
3. Sometimes
4. Rarely
5. Never
4. I elicit questions from patients using a patient-centered, open-ended approach (e.g., “what questions do you have?” rather than “do you have any questions?”)
1. Always
2. Often
3. Sometimes
4. Rarely
5. Never
5. I put the responsibility of clear communication on myself as the clinician (e.g., “I've just said a lot of things. To make sure I did a good job and explained things clearly, can you describe to me….?”)
1. Always
2. Often
3. Sometimes
4. Rarely
5. Never
